# Mothering on the edge: exploring maternal anger through feminist psychoanalysis and socio-ecological inequities of two mothers in urban India

**DOI:** 10.3389/fgwh.2026.1751532

**Published:** 2026-04-17

**Authors:** Ketoki Mazumdar, Praachint Kour

**Affiliations:** 1 Department of Psychology, FLAME University, Pune, India; 2 Department of Psychology, FLAME University, Pune, India

**Keywords:** feminist psychoanalysis, India, interpretive phenomenological analysis (IPA), maternal anger, qualitative research

## Abstract

**Introduction:**

Maternal anger, an intense and often stigmatized emotional experience, has been associated with postpartum mood and anxiety disorders. This biomedical lens obscures its relational and sociocultural roots and remains understudied in psychological and sociological literature, especially from a qualitative stance contextualised in an LMIC. Drawing on Benjamin's feminist psychoanalytic theory and Bronfenbrenner's social-ecological model, this study explores maternal anger as a meaningful, biopsychosocial response to relational ruptures and systemic inequities in an Indian context.

**Methods:**

Vignettes of two urban Indian mothers from diverse backgrounds were chosen to present their lived experiences with maternal anger. Their in-depth, semi-structured interviews were analyzed using the Interpretive Phenomenological Approach.

**Results:**

A relational-intersubjective lens guided attention to mutual recognition/ruptures and affective attunement/misattunement, while the social-ecological model mapped influences across the nested systems. The first theme that emerged was Experience of Anger in Relational Qualms, which included the sub-themes of Betrayal and Abandonment in Intimate Relationships and Erasure of their Personhood. The second theme, Socio-cultural Experience of Anger, consisted of Emotional Burden of the Care and Lack of Support, Suppression and Silence, and Intergenerational Scripts.

**Conclusion:**

Reconceptualising maternal anger at the intersection of interpersonal and systemic pressures reframes it from pathology to protest. It becomes an embodied critique of relational, gendered and systemic inequities and calls for perinatal mental health interventions that honor mothers’ emotional realities. By depathologizing maternal anger, the lived experiences of the two urban Indian mothers contribute novel insights to maternal mental health and underscore the necessity of feminist, context-sensitive approaches in research and care.

## 1 Introduction

### 1.1 Background

Maternal anger is often dismissed in psychiatric discourse as a symptom of postpartum “disorder,” a transient psychophysiological imbalance (1–6), primarily to be silenced (7, 8) in clinical and popular discourse. It is associated with a loss of emotional control and a persistent feeling of failure to be a mother (9). For several mothers worldwide, intense anger is not a pathology but a visceral response to systemic abandonment and misattuned relations. An experience against society's demanding selfless caregiving and nurturing while denying mothers the right to care for themselves (10–13). Intensive mothering is almost a normative standard (14). Rich's seminal distinction between motherhood as a patriarchal institution demanding selflessness and mothering as a complex, embodied experience underscores the tension between societal expectations of intensive mothering and maternal autonomy. Rich further argued that institutionalized motherhood reduces women to instinct-driven caregivers, erasing their selfhood (15), producing ambivalence, exhaustion and isolation that fuels maternal anger.

In India, these dynamics operate within intersecting systems of patriarchy, class, religion, and kinship. Studies show that women are socialized to suppress distress in the perinatal period, and vocalizing anger is often called out as selfish or socially disruptive (16, 17). Culturally, the “good mother” archetype in India valorizes self-sacrifice, demanding women prioritize familial needs over their own, and perpetuating cycles of guilt and silence (17). Such silencing, Rich (15) asserts, sustains heteronormative patriarchy by invisibilizing maternal anger. Nelson (18) in her 2015 piece noted that “a good mother is a happy mother” and “a sad mother is a bad mother”. Rapid urbanization, shrinking intergenerational support, and expanding paid work have amplified mothers’ time poverty and emotional burden.

Feminist scholars advocate for dismantling these oppressive narratives through collective storytelling. Sharing authentic, lived maternal experiences, embracing anger alongside joy, can forge solidarity, challenge institutionalized myths, and reclaim maternal subjectivity (15). Taking this forward, Iyer (16) rejects prescriptive ideals, asserting there is “no right or wrong” in mothering, urging people to open up spaces for diverse, unvarnished narratives. Maternal anger needs to be understood as emerging from structural and relational inequities that invisibilize maternal labour (9, 19–25). This phenomenon is also understood as socially inappropriate (26), as good mothers must constantly engage in emotional management (27) and serve as good role models for their children (26). Recent findings from the Time Use in India Survey (28) underscore a stark and persistent gendered scale of this burden. Women in urban India continue to spend close to six hours daily on unpaid domestic and caregiving work, in contrast to men's average of less than 90 min. In these contexts, maternal anger is shaped by both close-tie failures of recognition and broader socio-ecological pressures.

This paper uses two idiographic case vignettes from urban India to explore how mothers make sense of intense maternal anger. Drawing on Benjamin's relational intersubjectivity (29) and Bronfenbrenner's socio-ecological framework (30), we unpack anger as a biopsychosocial phenomenon that indicates interpersonal rupture. Centering mothers’ language and lived narratives allows us to connect phenomenology with context. It also allows us to provide access to culturally relevant care and consider implications for perinatal mental health.

#### 1.1.1 Problem statement—pathologization of maternal anger in psychiatry

A biomedical lens and psychiatric classification frequently subsume motherhood-related anger under diagnostic categories (e.g., postpartum depression, adjustment disorder) or reduce it to hormonal causes (31, 32). In documentation and coding, anger may appear as irritability or be placed under broad perinatal disorder codes (ICD-10-CM: O99.34; R45.4; F32.89; F43.20; Z65.8), a practice that risks decontextualising mothers’ experiences and narrowing responses to individual-level treatment (33). While anger may arise from threats to a mother's autonomy, disrespect, injustice, or broken norms (34), its expression is constrained by societal expectations that stigmatize women's emotional assertiveness. When anger becomes reduced to a diagnostic checkbox, its relational and structural roots are at a risk of being obscured—what feminist philosopher Silvia Federici (35, 36) terms the “social factory” of reproduction, where mothers’ bodies and labor are mined for capitalist, neoliberal, and patriarchal profit. For instance, a mother's anger at her partner's refusal to share caregiving could be labelled irritability. A feminist reframing does not dismiss the clinical lens but calls for a co-interpretive approach to reframesuch pathologization and instead recognizing anger as an understandable, embodied critique of socio-ecological inequities. This shift has direct implications for screening, risk assessment, and intervention design in resource-constrained settings where relational support and public services are often scarce.

#### 1.1.2 Maternal anger—existing evidence

Maternal anger has only recently received focused qualitative attention, yet existing studies (37, 38)—primarily from Western contexts—underscore its distinctiveness and sociocultural embeddedness. Potegal and Stemmler's (34) cross-disciplinary definitions frame anger as “a recalibrational emotion” responding to threats or injustices, situating maternal anger within a broader affective taxonomy. Scharp and Thomas (39) explored maternal identity and depression narratives, while Shields and Shields (40) theorized the social meanings of women's emotions. Ou et al.'s (38) grounded theory study of 20 Canadian mothers demonstrated that intense anger in the first two postpartum years arises from ‘violated expectations’ (e.g., infant sleep disruptions, unequal domestic labor) and ‘compromised needs’ (e.g., exhaustion, loss of autonomy). Importantly, half of those experiencing intense anger did not meet clinical criteria for depression, suggesting maternal anger as a discrete affective response to social conditions rather than merely a depressive symptom. Building on this, qualitative studies, mostly from high-income settings, document a consistent phenomenology of maternal anger—physiological arousal, intrusive harm imagery, loss of control, and post-episode guilt and identified key contributors such as child behavior, unmet personal and work-family needs, and pervasive stigma rooted in intensive mothering ideals (37). These experiences are contextualized within intensive mothering norms, which position mothers as self-sacrificing caregivers (10, 41).

Empirical studies on resource loss (42) and postpartum affective complexity (43) underscore that exhaustion and unmet needs fuel anger. Research on emotion management shows that gendered socialization frames anger as unfeminine and relationally threatening, which encourages suppression, anticipatory self-monitoring, and subsequent guilt and shame (26, 44, 45). Billotte Verhoff et al. (37) highlighted how mothers described ‘losing control’ during rage episodes, yet paradoxically restrained themselves from acting on violent visualizations (e.g., harming their child).

#### 1.1.3 Gap in non-western contexts

The tendency to medicalize maternal anger is amplified in collectivist, multigenerational settings such as India, where cultural norms and kinship structures shape how mothers’ emotions are experienced and judged. Much of the existing literature on maternal anger comes from nuclear-family contexts in high-income countries. This risks assuming family norms, welfare supports, and help-seeking patterns that do not map onto many Global South settings (46). In India, intergenerational scripts and joint-family dynamics may both support and police mothers, reproducing gendered labour expectations that compound time poverty and emotional invisibility (16, 17, 28, 47, 48). For many Indian women, visible expressions of maternal anger are often stigmatized, while private suppression becomes normative and acceptable.

Emerging data indicate that many Indian women report frequent anger in relation to paid labor, care burdens, and invalidation of their feelings (20, 49, 50). Adding to this complexity are phenomena such as the experience of enduring mental load management (23). These structural pressures interact with gendered socialization to produce patterns of private rumination, guarded expression, or episodic outbursts—all of which can be misread as pathological if the wider social context is ignored. There are limited empirical studies on maternal anger, especially locating it in the Indian context. The purpose of this study is to address this gap.

The tension between enforced silence and maternal anger is central to Indian feminist discourse. From early socialization, Indian women are taught to prioritize silence and respectability, vocalizing anger threatens their social standing (15, 16, 51). Modern Indian women navigate a paradox, participating in capitalist economies while adhering to traditional domestic expectations (52, 53). Despite employment, they remain secondary earners, burdened with unpaid caregiving (48), resulting in a recurrent dissonance, where intensive mothering ideals are practised in conditions with limited support, producing anger that is both an interpersonal signal and a critique of institutional failure. Yet, anger—whether vocal or suppressed—emerges as resistance to systemic erasure, disrupting the heteropatriarchal ideal of the “self-sacrificing mother” (47, 54).

There is limited qualitative research that locates maternal anger within these distinctly Indian socio-ecological dynamics; this study addresses that gap by offering idiographic, context-sensitive analyses. Duncan (55) notes that silence is culturally manufactured, not innate, a discourse that communicates subservience, as seen in Indian women's compliance with domestic roles. By reframing anger as resistance, feminist scholarship exposes the notions of idealized motherhood, where women's autonomy collapses under the weight of societal respectability (56, 57).

### 1.2 Theoretical framework

#### 1.2.1 Relational intersubjectivity and Feminist psychoanalysis: anger as rupture

This paper adopts two interdisciplinary lenses of Benjamin's Relational Intersubjectivity (29) and Bronfenbrenner's Social Ecological Model (30) to explore maternal anger. Together, they highlight how maternal anger emerges from failures in interpersonal recognition and structural inequities, particularly in urban India's rapidly shifting socio-cultural landscape.

Relational intersubjectivity theory posits that emotional experiences are co-constructed through dynamic, reciprocal interactions (29, 58). This means a person's sense of self and emotional experiences are developed through mutual recognition with others, rather than in isolation.

Mothering is deeply relational and an emotionally complex journey. Maternal anger often emerges when mutual recognition breaks down, such as when their labor and needs are invisibilized by partners, families, or institutions. Feminist psychoanalyst Jessica Benjamin's (29) concept of ‘doer-done to’ dynamics illustrates this. When mothers are placed in ‘done to’ roles (e.g., bearing sole responsibility for childcare), they experience anger as a protest against relational erasure; conflict arises when recognition fails, undermining mothers’ sense of agency and worth. From this perspective, maternal anger can be seen as arising from relational ruptures and is not simply an individual problem. Benjamin's (29) concept of mutual recognition is pivotal here, as when caregivers are denied acknowledgement as full subjects (e.g., mothers’ identity, labour and needs rendered invisible by her partner, family or even her child), relational ruptures occur, generating disconnection, anger, resentment and alienation. Winnicott's (59, 60) concept of the ‘holding environment’ adds that a ‘good-enough’ mother provides a reliably supportive context for the child's needs, implicitly suggesting that mothers too need a form of ‘holding’—recognition and care—from their close relationships. Without such support, the emotional demands of caregiving can become overwhelming.

When these relational processes align, mothers feel connected and valued. Psychologically, the mother's anger can be seen as an attempt to reclaim her subjectivity and assert that her experience matters, thus explaining how the emotional pain turns into anger. Feminist psychoanalysis extends this lens, critiquing how patriarchal systems enforce the “good mother” myth. Chodorow (61) argues that gendered caregiving reproduces maternal self-sacrifice. When mothers defy this ideal, possibly expressing anger at unequal domestic burdens or ruptured interpersonal and intrapersonal relationships, they face cultural gaslighting, abandonment, and stigma (51).

#### 1.2.2 Structural inequities: Bronfenbrenner's ecological model

Bronfenbrenner's ecological systems model (30) situates individual experience within multiple nested contexts, offering a scaffold to map how structural inequities amplify maternal anger in urban India. It highlights how a mother's emotions are influenced not only by her close relationships but also by broader societal forces. The model can be described as multiple layers (microsystem, mesosystem, exosystem, macrosystem, and chronosystem) that influence psychosocial positions. Collectively, these layers shape mothers’ emotional lives. All of these ecological factors (from immediate family dynamics to embedded cultural scripts) create a climate in which a mother's stress and suppressed anger can accumulate. Bronfenbrenner (30) emphasizes that development arises from reciprocal interactions between the person and the context.

At the microsystem level, mothers negotiate their most immediate social relationships—partner, child, extended family, where they experience direct support or lack of it, followed by failures of attunement. For example, a mother's anger may stem from her partner's ignorance or refusal to share childcare, reflecting Benjamin's (29) breakdown of recognition. Along with that, the data from recent survey data (28) point towards the stark imbalance of mothers spending up to six hours daily on unpaid care work in India, leading to chronic fatigue, emotional depletion, and a pervasive sense of isolation even within the home. All of this is a direct response to microsocial neglect.

The mesosystem captures the intersections between home and other domains—workspaces, healthcare, community, and shrinking extended-family networks in cities. Indian workplaces frequently lack flexible arrangements, forcing mothers into a precarious work–family tug-of-war. Long commutes in congested cities further drain emotional resources. The mesosystem thus becomes a crucible in which role-conflict intensifies, and maternal anger surfaces as frustration at being pulled in incompatible directions by inflexible institutions.

Mothers also inhabit exosystems which they do not directly control, such as government policies, neighbourhood resources, and media discourses. India's scant safe, and public childcare infrastructure and fragmented mental-health services leave mothers without institutional backup when relational support fails. Indian mothers in paid labor often face censure for abandoning domestic duties, exacerbating emotional strain, and are experienced as direct structural abandonment, fueling anger at a society that invisibilizes maternal labor.

At the macrosystem level lie the overarching cultural norms, ideologies, and socioeconomic structures. The ideal Indian mother valorizes self-sacrifice and silent endurance. An almost ’supermom’ figure who flawlessly balances career and family. Patriarchal discourses promulgate the myth of the ever-patient mother, rendering any expression of anger as deviance. Media, family expectations, and cultural symbols (e.g., multi-armed Hindu goddess Durga) reinforce the notion that multitasking is an inherent female ability. These cultural messages constitute a silent backdrop that tells mothers they should feel privileged to do it all and never complain. When mothers feel they must hide their anger or experience shaming, their anger intensifies as a form of protest against macrosocial erasure.

The chronosystem acknowledges that these layers shift over time. Rapid urbanization and the rise of nuclear families have eroded traditional support networks that once buffered maternal stress. Contemporary neoliberal reforms have dismantled welfare provisions, making support ever scarcer. In this evolving context, maternal anger becomes a reaction to everyday injustices along with a marker of historical change and loss of communal caregiving structures.

#### 1.2.3 Intersubjective ruptures in a collapsing ecology

A feminist psychoanalytic-intersubjective approach (29) foregrounds mothers’ subjectivity and emotional conflicts within relationships. Combining it with Bronfenbrenner's Socio-ecological theory (30) ensures these personal narratives are embedded in context. This shift is intentional as it reframes maternal anger from being an individual failure or purely pathological symptom to being a socially situated affect**.** Together, these frameworks reveal maternal anger as a biopsychosocial phenomenon, arising from the intersection of relational and ecological forces.

Maternal anger reflects ruptures in relational recognition and systemic neglect, where this dual lens allows us to depathologize anger. Such an interpretation paves the way for developing culturally supportive interventions and care that address both emotional needs and social conditions of mothers.

### 1.3 Research questions

By centering lived experiences, we reframe maternal anger as both a relational rupture and misattunement (the breakdown of mutual recognition in families and institutions) and a structural failure (a condemnation of policy failures and cultural gaslighting). Our analysis challenges the biomedical model's pathologisation of anger, arguing that anger must be reclaimed as an indicator of relational and structural injustice rather than a diagnostic category in itself.

#### 1.3.1 How does intersubjective ruptures and structural inequities shape mothers’ experiences of maternal anger in urban India?

#### 1.3.2 How can a feminist psychoanalytic depathologization of maternal anger inform a more just and responsive perinatal/maternal care practice in India?

## 2 Methodology

### 2.1 Participants and case selection

The paper focuses on two vignettes of urban Indian mothers with children below the age of ten. While one of them is a mother of two children, residing in a joint family in the western part of India, the other is a freelance working mother to a single newborn, living in a joint family set up, also from the western part of India. Typically, a patrilineal structure, Indian joint or extended families are ones in which two or more elementary families are joined together, characterized by multigenerational and multi-functional roles within it (62). They were selected to present their experience with maternal anger in the Indian socio-cultural context, as they presented an intersection of religion, culture, and professional/homemaker identity. Upon the institutional review board's (IRB) approval, they were recruited using a purposive and snowball sampling method using a call for participation poster, which was circulated on social media and other professional networking sites. The case presentations in this paper are a part of a larger study aimed at exploring the lived experiences of matrescence and maternal anger in urban Indian mothers.

This study employs Interpretative Phenomenological Analysis (IPA), an idiographic approach that privileges detailed, context-sensitive accounts to illuminate psychological processes (63). Our theoretical aim was to comprehend the processes and experiences of maternal anger and how it is co-constituted by failures of interpersonal recognition and by nested social systems. Accordingly, we integrated Jessica Benjamin's relational intersubjectivity with Bronfenbrenner's socio-ecological model. Selecting two richly descriptive cases is therefore a theoretically driven analytic strategy. Small, contrasting case sets allowed us to trace how similar affective phenomena, maternal anger, unfold differently across relationship dynamics and ecological contexts. We purposively selected the two exemplars from the larger project, using criteria that maximised theoretical leverage: (1) sustained first-person accounts of intense maternal anger, (2) transcript richness suitable for idiographic analysis, and (3) contrast on theoretically salient axes, such as religion, parity, region, reproductive history, and work role. One case is a Hindu mother from northern India with two children and a history of miscarriage, and the other is a Muslim freelance mother from western India with one infant—differences that enable close attention to how religion, parity, geographic setting, reproductive history, and occupational role shape recognition, intergenerational scripts, and access to supports. These two vignettes are presented idiographically to reveal depth and process; they are not intended as generalizable descriptions of all Indian mothers but as interpretive exemplars that illuminate relational and socio-ecological mechanisms of maternal anger. In line with IPA practice, we present detailed case-by-case analyses followed by cross-case abstraction to develop theoretical propositions about relational rupture and ecological neglect.

### 2.2 Materials and interview procedure

Participants expressed their interest in being interviewed by the researchers through the Google form that was circulated. The form collected basic socio-demographic details along with their consent for scheduling the interview. Interviews were set at a mutually convenient time through the form itself, ensuring their ease, access, and comfort with using time and technology. They were given detailed information about the study, its voluntary nature of participation, participant information sheet and the confidentiality aspects of the study. Written consent for audio and video recording was obtained, and their identities were pseudonymised as soon as the interviews were transcribed. Confidentiality was reiterated at the outset of the interviews.

After receiving the written informed consent, data were collected through semi-structured interviews that encouraged rich, lived conversations. The interviews were conducted online, using the Zoom video conferencing platform, in April 2025. Each session lasted for 60–90 min, where the participants were encouraged to share their emotional experiences with motherhood/mothering, with a particular focus on how maternal anger was experienced, along with the contributing factors. An interview guide was followed, where the mothers were asked open-ended questions such as, could you recall a moment/moments when you experienced anger in your motherhood journey? How do you typically express your anger in relation to your motherhood? The participants’ demographic details and background information about their childhood, present relationships, and current living arrangements were also collected in the interviews, which were conducted in English with some use of Hindi. They were video recorded, and the interviews were transcribed verbatim and stored safely in password-protected folders, which only the PI and Co-PI had access to.

### 2.3 Data analysis

The interviews were analyzed using the Interpretive Phenomenological Analysis (IPA) framework. This analysis offers a distinct avenue to delve into the layers of lived experiences of the participants, where the experience is expressed in its own terms rather than predetermined categories (64). Grounded in hermeneutics, the researchers attempted to understand the participants’ worldview and the language that shapes their experiences. By keeping the participant at the centre of inquiry, IPA becomes a dynamic process, where the researcher gains access to the participants’ experiences and further attempts to make meaningful connections to their personal world through the information provided by the participant (65). Each transcript was individually analysed to identify themes and subthemes. They were compared to draw out the commonalities and the differences between the cases. The analysis was done according to the structure provided by Smith et al. (63).

In the first stage, the transcripts were read again and again for the researchers to familiarize themselves with the data and to enter the participants’ world for active engagement. The second step included making initial exploratory notes based on free association, where the content of the transcripts was noted. This was done at various levels: descriptive, linguistic, and conceptual levels. On a descriptive level, the content of the interview was looked at, such as the keywords, specific phrases, and explanations that were used by the participants. This is done to understand the structure of the participant's thoughts and experiences. On a linguistic level, the use of language, which included the variations in tone, use of pauses, laughter, and metaphors, was considered, along with deciphering the meaning behind them. Finally, on a conceptual level, the researchers’ critical reflections and interpretations were integrated to provide a deeper and layered understanding of what was stated by the participants. The third step included making experiential statements that crystallized and consolidated the researchers’ thoughts, and reduced the volume of detail while maintaining the complexity of the data presented. This also helped in finding the emergent themes and the sub-themes for each participant. The researchers then carried out the same procedure for the second participant. The researchers ensured data trustworthiness by engaging in investigator triangulation and theoretical triangulation. While the investigator triangulation was done by both the researchers analyzing the data through the steps listed above, theoretical triangulation was done by employing multiple theoretical orientations to understand and direct the findings (66). When both transcripts were completed analyzing, the researchers found a number of themes that were identified across the sample that succinctly captured the participants’ experiences. The themes are discussed, and the conclusions drawn are resented henceforth.

**Figure 1:**
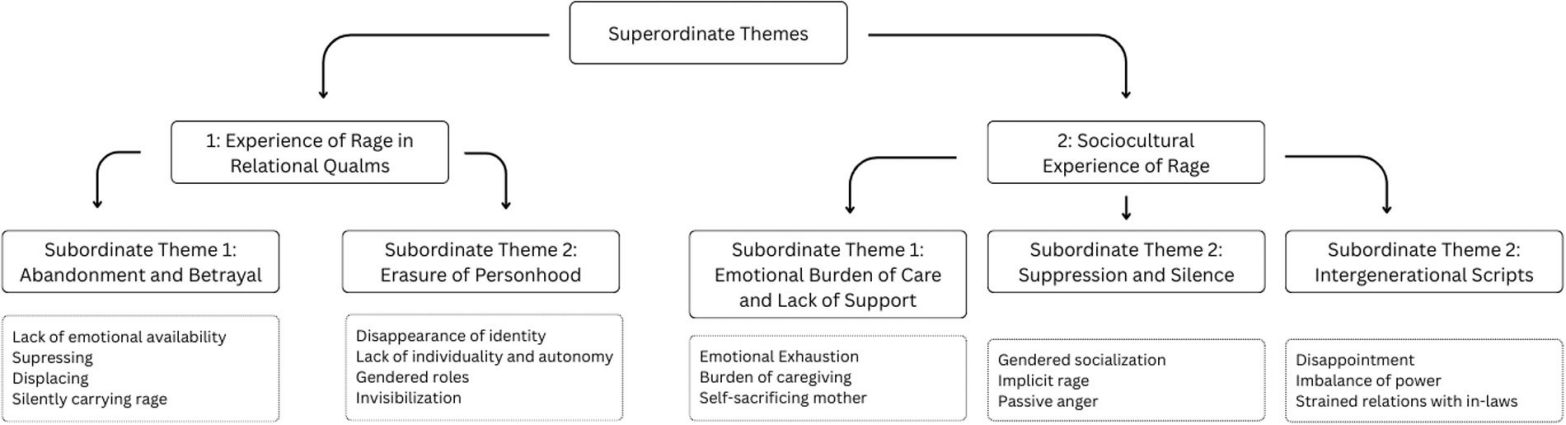
Illustrates the hierarchical structure of the themes developed through interpretative phenomenological analysis.

The analytic process was iterative and inductive, with ongoing movement between individual cases and cross-case patterns, consistent with IPA's idiographic and interpretive commitments. Reflexive memo-writing was used throughout the analysis to document interpretive decisions, emotional responses, and evolving theoretical insights. These memos supported analytic transparency and allowed ongoing critical engagement with how the researchers’ theoretical commitments and positionalities shaped interpretation, consistent with IPA's interpretive epistemology. To enhance analytic transparency, an IPA process table, Table 1 and a flow chart of the thematic process, Figure 1, are provided to explicitly show how verbatim excerpts were transformed into codes, experiential statements, and superordinate themes.

**Table 1:** Is an overview of the step-by-step utilized in interpretative phenomenological analysis (IPA), illustrating the sequential steps from initial data immersion to development of emergent themes and cross-case analysis.

Step	Process	Description	Example
Step 1	Familiarizing with data	Re-reading the verbatim transcripts and listening to the audio of the interview.	–
Step 2	Immersion of data	Initial note making, observations, including reflections on the interview along with any thoughts or comments. Focus on context, language, and initial interpretative comments.	*“I mean, at the same time there was a lot of anger because I wanted my husband to talk to her (Mother-in-law) about it (to help in household chores), you know, just at least give it a try, but he's like, ‘I know that it's not going to happen, so I'm not even interested in trying.’”* Descriptive: Participant expresses anger and frustration, desire for her husband's support and a sense of being alone. Linguistic: ‘*you know*’ indicative of seeking validation. Fragmented structure of sentences indicate emotional intensity. Conceptual: imbalance in emotional labour, unpaid household chores, powerlessness within her relationship, and relational invalidation.
Step 3	Identifying emergent themes	Crystallizing and consolidating the researcher's thoughts into emerging themes.	Unmet spousal support needs Invalidated concerns Silencing through refusal Internalized disappointment Relational disconnection Desire for shared responsibility Imbalance of labour
Step 4	Clustering themes	Emerging themes are grouped together to make connections and clusters, which gives superordinate and subordinate themes for each participant.	**Support** Unmet spousal support needs Desire for shared responsibility **Emotional Disconnection** Invalidated concerns Internalized disappointment Relational disconnection **Sociocultural Experience of Anger** Silencing through refusal Imbalance of labour
Step 5	Repeating step 1–4 for each participant	The researcher repeats step 1 to 4, by trying to bracket the previous themes, in order to do justice to the individual cases.	–
Step 6	Across case patterns	After individual analysis of each case, patterns of shared higher order qualities across are found, while noting idiosyncratic instances.	Superordinate theme: Experience of Rage in Relational Qualms Subordinate theme: Betrayal and Abandonment in Intimate Relationships Participants: P1 and P2 Convergence: Both the participants expressed desire for partner's involvement Divergence: P1 expressed disappointment with mother-in-law, due to the expectations set from her own mother. Moreover expressed intense anger. P2 expressed loneliness, blame and guilt for not being understood. Expressed passive anger, and active sadness.
Step 7	Deepening interpretations	The analysis is further deepened using metaphors and temporal referents, by utilizing theories as a lens to view the analysis.	Incorporating Bronfenbrenner's socioecological model and Benjamin's relational intersubjectivity to understand the experience of anger for the two mothers.

**Figure 2:**
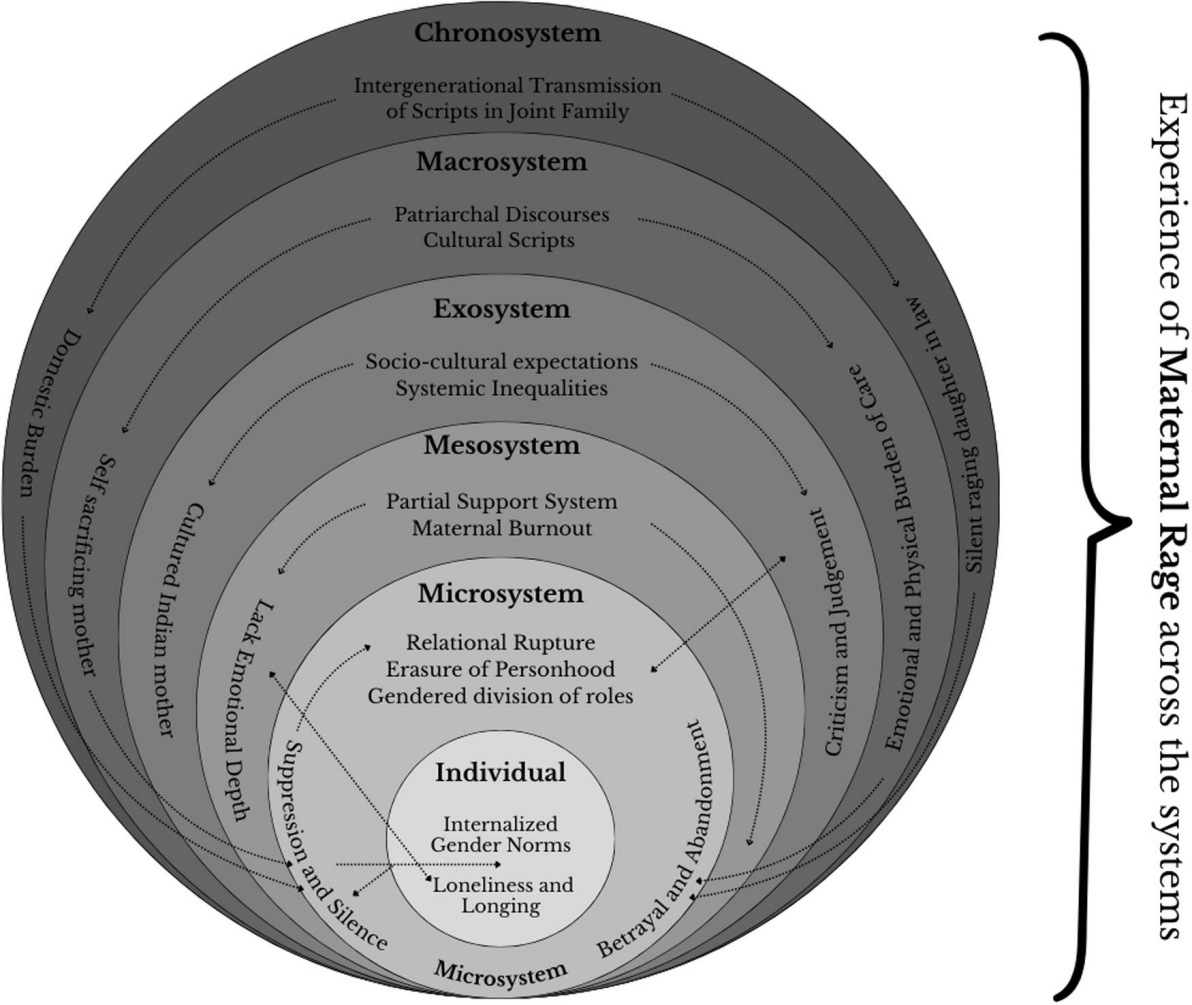
Illustrates the multilayered contributors to the experience of maternal anger using the socio-ecological model.

Given the idiographic commitment of IPA, the aim of our analysis was on depth, rather than breadth of exploration. We determined that the idiographic depth was reached when no new experiential themes, meaning or interpretative insights were being derived from the respondent's accounts in response to the research question. At that point, no novel conceptual understandings were being derived, indicating sufficient depth of analysis was achieved within the small, purposive sample that was selected. Moreover, apparent contractions in the respondents’ accounts were treated as analytically meaningful, where we examined the divergences closely by situating them within the broader narrative and contextual data. They were integrated into the analysis to indicate the complexity of the lived experiences of the respondents. This, along with the arrival of the superordinate themes have been integrated into the steps table.

Member checking was not performed, as the aim of this presentation through the IPA lens was to develop an interpretive account of participants’ lived experiences rather than to seek validation of analytic interpretations. IPA acknowledges the researcher's active role in meaning-making through a double hermeneutic process (63). To enhance analytic rigor and credibility, the study instead employed prolonged engagement with the data, reflexive memo-writing, and a transparent analytic audit trail. Two supplementary tables have been included to illustrate how verbatim excerpts were developed into experiential statements and superordinate themes.

### 2.4 Positionality statement

The first author identifies as a perinatal psychologist, intersectional feminist researcher, and primary caregiver with lived experience and proximity to the emotional and structural tensions of motherhood. She approaches this study from a lens of both personal and professional entanglements. Her social stance—as an upper-caste, urban Indian woman with academic privilege—shapes her access to language, institutions, and analytic tools. At the same time, she is deeply aware that such privileges coexist with the affective burdens of maternal labor, which remain largely invisible and normalized in everyday life. This research is grounded in a feminist, psychoanalytic, and critical psychological epistemology. The first author is committed to centering mothers’ voices not as data points, but as co-constructors of knowledge and meaning-makers whose voices and anger must be heard. She considers frameworks that isolate maternal anger as a clinical deviation, and proposes comprehending it as a relational and political response. Throughout this study, she has actively engaged reflexively with her role as researcher by attuning to silences, remaining accountable to the meanings shared in interview settings, and resisting the temptation to over-interpret their distress. This research is not detached from her own emotional investments; it is rooted in them.

The second author identifies as a brown, middle-class, unmarried, and childless woman. While her understanding does not come from the lived experience of motherhood, she has witnessed the experiences and stories of women around her, particularly her mother and blood relatives, where stories of sacrifice and silence have been embodied. Moreover, being a woman of colour born in a middle-class patriarchal family has allowed her to witness the socio-cultural narratives that shape womanhood, hence the resonance with some of the emergent themes of the study. Her position as an unmarried and childless woman working in the sector of mental health has allowed a critical distance that helps in noticing patterns that may be otherwise invisible and oppressive. Moreover, she was younger than the participants, which allowed her to approach the subject with curiosity and humility. While she was aware of her own embodied experiences of generational and gendered differences, her younger age helped her to listen with attentiveness and a newness to being submerged in the realities of motherhood.

### 2.5 Case vignettes

Preeti, a 40-year-old mother of two, lived in a joint family. She was a homemaker by choice and was deeply attached to her natal family. She married at the age of 25 and had her first child six years later. Her second child was born after a gap of three years. Preeti shared a close, cordial relationship with her husband; her relationship with her mother-in-law was strained.

Her husband supported her with some gendered childcare responsibilities, which were often laden by judgement and criticism towards the husband-wife dynamic. Moreover, she noted, in moments that she needed support with domestic chores and childcare responsibilities, none was offered to her, leaving her feeling isolated and emotionally exhausted. The transition into motherhood brought upon Preeti the expectations of being a ‘perfect’ mother who raised ‘perfect’ children. Over time, they built up her frustration and aggression, leading to ruptures in her interpersonal relationships.

Sana is a 29-year-old mother of an 8-month-old daughter. She was a working professional who scheduled and organized her work according to her newborn's schedule. She was also solely responsible for the domestic chores. She lived with her husband, daughter and her mother-in-law. While she shared a close relationship with her husband, her relationship with her mother-in-law was strained. After giving birth, she received support from her workplace; however, she was met with rejection and dismissal from her husband and her mother-in-law when it came to sharing responsibilities. Hence, she spent most of her daytime alone with her child, managing childcare and domestic responsibilities, with no one to talk to, or with no moment to herself. This built-up frustration and irritation towards her family. Sana drew parallels with how her mother had treated her after giving birth, which clouded her expectations of her mother-in-law. She described her pregnancy and motherhood journey as one of the most difficult phases. She felt lonely and developed a sense of resentment in her experience of motherhood.

These vignettes provide contextual grounding for the idiographic analyses that are going to be presented below. It allows examination of how maternal anger was experienced, interpreted, and negotiated within each participant's relational and socio-ecological context, consistent with IPA's case-centred analytic approach.

## 3 Findings

### 3.1 Idiographic case analyses

#### 3.1.1 Preeti: anger as rupture, recognition-seeking, and moral injury

For Preeti, maternal anger emerged within a relational environment characterised by chronic misrecognition and emotional invalidation. Her narratives revealed that anger was not an impulsive or irrational state, but rather a response to persistent experiences of being unseen, judged, and emotionally unsupported. While she fulfilled extensive caregiving responsibilities, her efforts remained unacknowledged, producing a dissonance between her internal experience of sacrifice and the external perception of her as inadequate. Her statement that others “only see you hitting the child, but not why you are sad” illustrates this rupture in mutual recognition, where her distress was reduced to observable behaviour rather than understood as a relational signal.

Preeti's anger thus functioned as a communicative affect—a response to what Benjamin (29) conceptualizes as the breakdown of recognition. Yet, rather than eliciting repair, her anger was further moralized and pathologized, reinforcing feelings of shame, abandonment, and isolation. Over time, this contributed to an erosion of her sense of personhood, as she internalized expectations of being a self-sacrificing mother while suppressing her own emotional needs. Her anger oscillated between outward expression and inward containment, reflecting the tension between her need for recognition and the cultural mandate to remain emotionally restrained.

Ecologically, Preeti's experience was shaped by multiple nested systems. At the microsystem level, strained relationships with her mother-in-law and inconsistent emotional support from her husband created relational vulnerability. At the macrosystem level, intensive mothering ideals and patriarchal expectations reinforced her emotional silencing. Thus, Preeti's maternal anger emerged not as individual pathology, but as a relational and ecological response to chronic emotional invisibility and structural inequity.

#### 3.1.2 Sana: anger as silent endurance, emotional withdrawal, and relational detachment

In contrast to Preeti's episodic outward expressions, Sana's maternal anger manifested primarily through withdrawal, emotional detachment, and private containment. Her narratives revealed anger as a chronic, quietly accumulating affect shaped by persistent isolation and relational abandonment. Despite her expectations of shared caregiving, she encountered repeated dismissal from her husband and mother-in-law, leading to a gradual erosion of relational trust. Her husband's statement, “don't expect anything”, symbolised a withdrawal of relational responsibility, forcing Sana into a position of solitary caregiving.

Unlike Preeti, whose anger occasionally surfaced visibly, Sana's anger was largely internalised. She described documenting her experiences privately, indicating a need to witness and validate her own emotional reality in the absence of relational recognition. This act of private witnessing functioned as both a coping mechanism and a form of self-preservation, allowing her to maintain relational harmony while containing emotional distress. Her anger, therefore, existed as a muted, enduring presence—less visible, but equally profound.

From a socio-ecological perspective, Sana's anger was shaped by intersecting pressures of gendered caregiving expectations, joint-family hierarchies, and the systemic absence of relational support. While her workplace provided structural accommodation, her immediate relational environment failed to provide emotional attunement. This mismatch between institutional and interpersonal support intensified her experience of loneliness and emotional abandonment. Thus, Sana's maternal anger emerged not as episodic rupture but as sustained relational deprivation. Her experience illustrates how maternal anger can exist as quiet endurance—a prolonged response to emotional neglect and structural inequity—rather than overt emotional expression.

The following sub-section is going to present two themes that emerged from the two idiographic vignettes: (1) Experience of Anger in Relational Qualms and (2) Socio-cultural Experience of Anger. Each theme comprises sub-themes that show how interpersonal ruptures and socio-ecological forces co-produce maternal anger in everyday life.

### 3.2 Experience of anger in relational qualms

This theme focuses on how the mothers’ closest relationships—partners, in-laws, and household networks—shaped the emergence, tone, and aftermath of their anger. Anger here was rarely a single event; it accumulated as nights of exhaustion, repeated invisible slights, and persistent de- recognition. It outlined how their relational experiences caused a rupture, further affecting their inner world.

#### 3.2.1 Betrayal and abandonment in intimate relationships

Both Preeti and Sana described anger that was rooted in their maternal roles and interpersonal and relational dynamics in repeated patterns of betrayal and abandonment, rather than a single trigger, where any expression of seeking help was denied. Beneath the dominant cultural expectation to do it all, they were also expected to carry the emotional burden without any complaints.

For Preeti, anger often followed a day of feeling unseen—the household noticing her child's behaviour but not the exhaustion or humiliation that preceded it. In her words: “*When I used to hit my child, my brothers used to go crazy that I hit them, but nobody gave a damn that why do I hit him? Why am I sad?’ — they only saw the act, not the pain behind it.*” That sense of being judged for an outcome rather than understood for the cause repeatedly eroded her sense of agency and produced episodes of intense shame and inward anger. There was a strong desire to be seen as a human being; however, her family often disregarded her emotional experiences and criticized her harshly. While there was a deep longing to be understood, she also indicated her sense of abandonment was felt because of the loneliness of carrying both the guilt and the emotional weight of her actions, “..*everybody takes a back seat, and everybody will… say I am like this, I only don't want to change.”*

Sana's anger, by contrast, was quieter but equally corrosive. Her husband's repeated minimization—“*don't expect anything*”—functioned as a withdrawal of recognition. It normalized his absence and almost licensed her solitary caregiving. Further, her betrayal and abandonment were experienced more intensely with her mother-in-law, too. This withdrawal was not always dramatic; rather, it operated as an ongoing background that left Sana with chronic loneliness and a deep resentment. Expectations towards her mother-in-law, towards helping her, came to rise due to her own experience with her mother, who saw and supported her in the early days of her motherhood. Her needs were often met with rejection from a potential source of maternal relatedness and became a site for relational rupture in the form of betrayal and intense emotional pain at the microsystemic level. “*I thought more than anybody, my in-laws would be elated, but it has really turned exactly the opposite..I was pretty shocked and very surprised because I knew how badly my in-laws wanted a grandchild. And now that the grandchild is here, you refuse to even look at her*”. She captured the experience of muted fury and practical relational collapse when she said, “I have to do what I have to do. I have to get on with my work,” a remark that revealed how gendered expectations framed her time and choices.

Together, these narratives show how anger functioned as a communicative signal, at the microsystem—a demand for recognition—even when it was expressed reluctantly or contained. Preeti loses her temper with her child after a day of feeling unseen by her spouse and misunderstood by her family, and Sana navigates the anger passively on her own**.** Where support was refused or minimized, both mothers experienced relational rupture; the emotion registered not only as intrapsychic distress but as evidence of relational failure. In isolation, this is a relational event; yet it occurs under the macrosystem's watchful eyes. Without institutional support at the macrosystem and exosystem, both mothers have few practical options to relieve pressure, so their resentment and isolation build further, adding fuel to their fury.

#### 3.2.2 Erasure of their personhood

A second recurrent pattern was the gradual erasure of personal identity after motherhood. Both mothers described how roles and expectations narrowed their personhood into serviceable functions (mother, daughter-in-law), leaving little room for individual wants or small freedoms Preeti described the transition into a new family, where she experienced persistent criticism about her individuality and her mothering practices: “*You come to a different family … their mindset is totally different, and you are judged … you don't know how to behave, you're overspoken, or you're over social, always laughing*”. This conveyed how social demands of an ideal daughter-in-law and mother alter habitual self-expression. For Preeti, this relational and personal misattunement and rupture transitioned into internalized shame and anger that turned inward.

Sana's account emphasized the slow stripping away of agency; even small acts like going for a short walk were policed and declined by family members. She described practical restrictions and the loss of personal time in a way that made her feel invisible as a person outside her caregiving role, “*Only for an hour in the morning, you keep the baby, I'll just go for a walk. I won't go very far…only so that in case something happens, I can just run and come back. So that is also something that my mother-in-law was not agreeable to…she said it happens when you deliver, you tend to become healthy and stuff like that,*” she said, expressing regret that domestic labor eroded opportunities for intimacy rather than enhancing them. This quiet depletion of self—the slow attrition of autonomy—produced a distinctive, simmering anger; not explosive but steadily corrosive. This breakdown of the woman, without any support systems to lean on, coupled with a lack of recognition for who she was, enabled a quiet anger that bubbled within her.

The notion of mutual recognition parallels the idea of reciprocity, where both imply that mothers and their settings shape each other. This section recognized that both the mothers’ unresolved identity conflicts and relational dynamics played out against structural pressures, such as patriarchal gender roles, isolation, and invisibilization, in producing anger. In other words, maternal anger was experienced by Preeti and Sana at the intersection of interpersonal dynamics and sociocultural stressors.

### 3.3 Socio-cultural experience of anger

The theme situates the mothers’ affective lives in wider social patterns—cultural norms, institutional gaps, and intergenerational scripts—that shaped how anger could be felt, expressed, or contained. It outlined how the affective experience is embedded in the socio- cultural context, leaving little room for the expression of anger.

#### 3.3.1 Emotional burden of the care and lack of support

Both Preeti and Sana described chronic emotional exhaustion produced by an unequal and unrelenting care burden without any additional support. They were a part of the dominant cultural narrative of the traditional self-sacrificing mother, who was expected to carry the weight of domestic duties without any rest or recognition, leading to suppressed anger, a deep sense of bitterness and emotional isolation.

Preeti expressed her painful experience, “*I was expected to run my course as they are supposed to be. And even if my son wakes up at 5, she (Preeti's MIL) used to expect all the household work to be completed, but she never would offer help. I was constantly expected to do that*.

*Even to put the child in the pram, keep the child with me in the kitchen and work. As in no help.*” where she was not only denied any help, but there was an undertone that was laden with the expectation of *performing*, not just for the child, but also for the rest of the family. This invisibility and lack of care were internalized as someone who became a silent sufferer, with a buildup of resentment towards everyone, with no outlet for expression. This helplessness indicated suppressed anger towards the family.

Sana's account highlights the time-poverty dimension. Her domestic tasks consumed hours she wished she could have spent with her infant, producing regret that fed a wider anger at a system that normalises invisible labour. Her private refrain, “*my mind is racing… I need to get this and that done”*, turns practical overload into a felt loss of intimacy and agency. She was solely seen as a caregiver who had internalized the constant need to do everything. This, in turn, affected her presence in her newborn's life, where “*a recurring feeling that I always have is that I wasted a lot of time that I could have spent with my daughter.*” Sana indicated a deep sense of regret, which was weighed down by repressed anger. It also reflected a larger anger at the system for not supporting mothers, for celebrating invisibility, and for normalizing the time lost. Hence, this anger became an isolating experience that pushed her away from her support systems, causing ruptures and strain in the ecosystem.

The expectations placed on Preeti and Sana further indicated how emotional and physical labour was extracted from them under the guise of responsibility, leaving them raging internally and in isolation. Ruptures were experienced at the microsystem level—their intimate relationships. The absence of emotional understanding and empathy at the microsystem created a disconnect.

#### 3.3.2 Suppression and silence

Gendered socialization shaped how both Preeti and Sana carried their maternal anger; neither expressed their anger openly; both learned to contain it in their own implicit ways. This form of anger protected them from their harsh and rather painful reality, leading to a quiet withdrawal from intimate relationships. This indicated a conditioned response rooted in the patriarchal setup, learnt from the cultural values and beliefs, that women, specifically mothers, are not allowed to voice their opinions.

Preeti put it plainly, “*I will not, you know, get into the argument. I will just detach.*” Detachment preserved surface harmony and went with the ideal of a *good Indian mother*, who does not rebel. Moreover, her silence was also a way to protect her relationship with her husband, which was characterized by fear and isolation. This accreted, and she expressed, “*Somewhere it stays in your heart*”, describing a quiet store of anger that eroded her sense of self. This silencing, which is experienced in isolation and without any emotional support, makes the anger quiet, unvoiced, and yet deeply felt, rupturing the mother at her most vulnerable moments. Once again, this happens in isolation, albeit under the watchful eyes of the nested systems that she is navigating daily. In this sense, it is felt as a self-dejection that makes her feel that she is rather tied to these larger ideas and beliefs, further suppressing her internal anger.

Sana's containment took a different form. Repeated emotional dismissal by her husband and mother-in-law made confrontation feel futile, so she turned to private witnessing of her anger. “*What I’ve started doing is actually jot down my experiences about what happened at what time…there's a party at home…I become very sensitive about who's helping me out,*” she explained. Journalling gave her agency, and a record of hurts, but private containment also converted anger into rumination and long-term mistrust, “*I may forgive, but I will definitely not forget*”. This represented the dominant cultural narrative of a mother's anger and distress that is often muted, silenced, and managed internally.

From a socio-ecological perspective, suppression is produced by microsystemic invalidation, such as partners, kin and macrosystem expectations, such as the “good mother” script. These containment strategies reduce visible conflicts but increase isolation and unresolved distress; silence therefore functions as both coping and constraint—a relational manoeuvre that masks deeper needs for recognition and repair. Hence, their anger becomes a multilayered experience that is deeply personal, shaped by social, cultural, and political structures.

#### 3.3.3 Intergenerational scripts

Through analysis, mother-in-law/daughter-in-law dynamics repeatedly surfaced as sites of expectation, comparison, and emotional erasure for Preeti and Sana. Both of them had hoped for practical and emotional support from elder kin, but instead encountered judgment and policing of behaviour.

Preeti described being measured against family norms and dismissed, “she was like the typical mother-in-law, she was never fond of me or something, or she was always into comparison, she never gave a damn,” a line that captures how comparison and disdain reproduce feelings of rejection. For her, such interactions reinforced the sense that her labour and distress were invisible and morally judged. Moreover, the use of ‘typical mother-in-law’ indicated the larger socio-cultural script of the hierarchical nature of family systems, where neglect and power imbalance were prominent. The narrative she heard was “*we are not here to offer you help*”. Due to the overbearing nature of the emotional and physical labour, it had a ripple effect, where feelings of isolation were amplified, “*It's like when your child, and your life is over*”, perpetuated by the macrosystem.

Sana articulated a resigned acceptance that masked pain: “*I don't have any expectations; if my mother-in-law offers, that's very good. If not, I know I’ll do it.*” Her comment signals protective detachment—a strategy to avoid the recurring hurt of unmet expectations—but also illustrates how intergenerational silence perpetuates cycles of unpaid labour and emotional neglect, leading to frustration and a silent anger. Sana further shared, “*What also makes me really upset is, you know, not only my mother-in-law, but any mother-in-law was also a mother, right? She was also once 30 years old, and she also once had one baby. And, you know, maybe it may have been so isolating for her also. And after all these years, you just come back and do the same thing to me”*. This also indicated an absence of a nurturing maternal figure who might otherwise offer emotional support, which perpetuated the joint family system, where the same expectations and roles continued in new forms, without altering the dominant discourse. Both narratives show that intergenerational scripts transmit gendered caregiving norms; rather than alleviating burden, they often reproduce it, producing quiet anger that is at once personal and systemic. Their anger, rooted in grief, was characterized by contradictory feelings of wanting to belong, yet feeling excluded. Intergenerational anger is not only relational but also a systemic concern that tightly holds the women in the cultural script of oppression and patriarchy.

## 4 Discussion

This idiographic IPA study demonstrates that maternal anger among urban Indian mothers is not merely a transient symptom of postpartum mood disorder but a meaningful, embodied response to relational misrecognition and layered socio-ecological failures. Using two detailed case vignettes, we identify two complementary phenomenological patterns: (a) episodic, visible anger that surfaces where moral judgement and shame follow overt expressions (Preeti), and (b) quiet, enduring anger contained through withdrawal and private witnessing (Sana). Both patterns are organised around the same core dynamics—continuous invisibilization of labour, unmet relational recognition, and constrained autonomy—but they differ in their modal expression and relational consequences. These affective forms often occur in the absence of diagnosable depression, indicating that maternal anger can be a discrete affective response to social conditions rather than simply a depressive symptom (37, 38).

Conceptually, the findings deepen and extend two theoretical frames. First, through a relational-intersubjective lens (29), anger is best read as a signal of failed mutual recognition: mothers used anger to assert subjectivity when household interactions erased their personhood, and yet that same anger was frequently moralised, producing shame rather than reparative recognition. Second, embedding these cases within Bronfenbrenner's socio-ecological model (30) shows how microsystem ruptures (partner and in-law disengagement) are amplified by mesosystem constraints (workplace inflexibility, fragmented support) and macrosystem norms (intensive-mothering ideals, patriarchal expectations). In doing so, we shift the analytic focus from intrapsychic pathology to relational-ecological process: anger both indexes interpersonal injury and signals upstream structural deficits (29, 35, 36). Methodologically, the juxtaposition of two contrasting idiographic cases demonstrates how the same social pressures produce divergent affective strategies (outburst vs. containment), thereby refining the view that anger principally functions as a communicative call—here it can also be a protective, self-preserving stance.

The study localises maternal anger within features that are salient in many LMIC contexts: joint-family expectations, intergenerational power hierarchies, acute time poverty, and limited institutional childcare. Urban Indian mothers navigate simultaneous pressures—persistent unpaid caregiving, shrinking intergenerational support, and neoliberal work demands—that produce chronic strain (28, 48). Intergenerational scripts (mother-in-law policing, duties of the daughter-in-law) can reproduce rather than alleviate labour burdens, converting what might be social support into an axis of policing and invisibilization (67–73). In resource-constrained settings where perinatal services and social protections are thin, reading anger only through a psychiatric lens risks misdiagnosis and missed opportunities for relational and policy remedies.

### 4.1 Implications for practice, policy, and research

The findings of this study have important implications for perinatal mental health care, particularly in resource-constrained settings such as India, where relational and structural determinants of maternal distress often remain unaddressed. Clinically, maternal anger should be understood not solely as a symptom requiring psychiatric classification but as a relational signal that may indicate unmet emotional needs, unequal care distribution, and structural strain. Perinatal health providers—including obstetricians, mental health professionals, and community health workers—would benefit from expanding screening practices to include relational and contextual dimensions of distress. This may involve assessing perceived emotional recognition, caregiving burden, relational dynamics with partners and extended family, and access to material and social support. Recognizing anger as an intelligible response to relational inequity can enable clinicians to move beyond symptom reduction toward interventions that support relational repair, such as couple-based attunement work, family mediation, and peer-support models that provide mothers with spaces for recognition and shared meaning-making.

At a programmatic and policy level, these findings highlight the need to integrate relational and psychosocial dimensions of maternal mental health into existing maternal and child health systems. National initiatives such as the Janani Suraksha Yojana (JSY), POSHAN Abhiyan, Reproductive, Maternal, Newborn, Child, and Adolescent Health (RMNCH + A), Pradhan Mantri Surakshit Matritva Abhiyan (PMSMA) and the National Mental Health Programme have significantly expanded access to maternal health services but continue to prioritize biomedical and nutritional outcomes, with limited attention to relational wellbeing. Embedding maternal mental health screening and relational support within these platforms offers a scalable pathway for addressing maternal distress. Community health workers, including Accredited Social Health Activists (ASHAs) and Anganwadi workers, are uniquely positioned to identify early signs of relational strain and maternal distress and to facilitate access to psychosocial resources, peer-support groups, and referral pathways. Structural supports, including accessible childcare services, workplace flexibility, and policies that encourage shared caregiving responsibilities, are equally critical, as they address upstream conditions that contribute to chronic maternal distress.

These findings also point to important directions for future research. There is a need for culturally grounded measures that capture the relational and socio-ecological dimensions of maternal anger, particularly in non-Western contexts. Future work should examine how maternal anger unfolds across diverse socio-economic, cultural, and family contexts, and explore how relational and community-based interventions can mitigate distress. Longitudinal and mixed-methods studies would be particularly valuable in understanding how maternal anger evolves over time, how mothers navigate relational repair, and how structural supports shape maternal well-being.

Taken together, these implications underscore the importance of shifting from an exclusively biomedical model of perinatal mental health toward a relational and socio-ecological framework that recognizes maternal anger as a meaningful response to lived conditions. Such a shift is especially critical in resource-constrained settings, where addressing structural inequities, strengthening relational supports, and expanding community-based care can play a transformative role in improving maternal mental health outcomes. These reforms would advance perinatal mental health in ways that align with Sustainable Development Goal 3 (health and well-being) and Goal 5 (gender equality).

### 4.2 Strengths and limitations

This study is perhaps among the first to explore the lived experiences of maternal anger within the urban Indian context.. The authors feel the strengths lie in its rigorous centering of lived experiences, which humanizes maternal anger by amplifying the voices of urban Indian mothers who often get muted in dominant sociocultural discourses.

Through two richly detailed case vignettes, it bridges the personal and political, employing an intersectional socio-ecological and relational framework to unveil how anger emerges from both relational ruptures, e.g., familial misattunement and structural inequities, e.g., gendered caregiving mandates, and invisibilization of their sense of self. By anchoring the study's analysis in India's heteropatriarchal context, the study fills a critical gap in perinatal scholarship, which has historically privileged Western perspectives. It also challenges pathologizing narratives through a feminist psychoanalytic lens that reframes anger as sociopolitical resistance. The methodological richness of case studies encapsulates the interpersonal and systemic complexities of motherhood, offering nuanced insights drowned by quantitative approaches. Theoretically, it innovates by integrating relational intersubjectivity with structural critique, illustrating how a psychoanalytic lens can decipher systemic interactions. Beyond academia, the study's praxis- oriented insights, such as advocating for compassionate perinatal care and community-rooted support networks, hold transformative potential, empowering mothers to reclaim their narratives and fostering solidarity against oppressive sociocultural norms. Ultimately, it positions maternal anger not as an individual failure but as a collective diagnosis of societal inequity.

While the study offers critical insights, its limitations must be acknowledged. The reliance on two case vignettes, though rich and in-depth, cannot fully represent India's diverse maternal populations, which limits transferability, as the experiences of urban, middle-class mothers cannot fully represent India's socioeconomically, regionally, and culturally diverse maternal populations. The analysis, rooted in traditional heteropatriarchal norms and urban contexts, may overlook nuances in rural settings or marginalized communities (e.g., Dalit, or queer mothers), where structural oppression intersects differently with motherhood. Additionally, the exclusion of multi-interlocuter perspectives (e.g., partners, healthcare providers, family members) limits a holistic understanding of how relational and systemic dynamics are co-constructed. These limitations, however, underscore the need for intersectional, longitudinal, and comparative future research to expand the discourse on maternal anger as a global feminist concern.

A further limitation is that the analysis is based solely on mothers’ accounts and does not include parallel perspectives from spouses or other family members. As an idiographic phenomenological study, the analytic focus was intentionally centered on mothers’ lived experiences and meaning-making rather than on corroborating relational dynamics across participants. However, including perspectives from partners, in-laws, or other household members could provide additional insight into relational attunement, caregiving negotiations, and structural constraints within the family system. Future research using multi-perspectival or family-based qualitative designs would help deepen understanding of how maternal anger is experienced, interpreted, and negotiated across relational contexts.

## Conclusion—adopting a justice-oriented approach

Reframing maternal anger from pathology to a signal reframes perinatal care, foregrounding recognition, redistribution, and institutional repair. Depathologising does not minimise risk or suffering; rather, it enlarges the array of remedies to include relational repair and social policy. In resource-constrained settings, attending simultaneously to household recognition and structural supports is essential if perinatal services are to reduce the conditions that produce sustained maternal anger and to promote maternal wellbeing more broadly. Such reforms would advance perinatal mental health in ways that align with Sustainable Development Goal 3 (health and well-being) and Goal 5 (gender equality).

## Data Availability

The raw data supporting the conclusions of this article will be made available by the authors, without undue reservation.
