# Supplementary material for: Mothering on the edge: exploring maternal anger through feminist psychoanalysis and socio-ecological inequities of two mothers in urban India
Source: Front Glob Womens Health. 2026 Apr 17;7:1751532. doi: 10.3389/fgwh.2026.1751532 (PMC13132850; doi:10.3389/fgwh.2026.1751532)
Supplement: Supplementary file 1 [file Table1.docx]

**1 Supplementary Table**

**1.1 Code Book Table 1**

| Theme | Description | Quote |
| --- | --- | --- |
| **Experience of Rage in Relational Qualms** | Mother’s relational rupture in her intimate relationships, that is shaped by gendered expectations, lack of acknowledgement and cultural conditioning. | *“I'm unnecessarily doing things alone when I when I'm actually not alone.”* |
| *Betrayal and Abandonment in Intimate Relationships* | Mother’s experiencing a sense of betrayal and abandonment as they were expected to carry the emotional and physical burden of caregiving and domestic chores. | *“I had my hopes on my mother-in-law. Why? Firstly? Because of the example, my own mother set and secondly, she's a lady, right? She's a woman.”* |
| *Erasure of their Personhood* | A slow and implicit erasure, by placing cultural norms and ideals along with expectations of motherhood on the mothers. | *“Lot many years I used to you know refrain from going out. Not for the, you know, I was like so involved in my kids, I was only happy being with them at home and hardly used to party, or socialize or go out.”* |
| **Socio-cultural Experience of Rage** | Mothers rage that is placed in structural inequalities, socio-cultural context and gendered expressions of affective experiences. | *“So, it's not that my mother-in-law was used to having this kind of help available, you know, like me doing everything. So when I was not there, she only used to do it…Now we have like a daughter-in-law so now she's just gonna do everything.”* |
| *Emotional Burden of Care and Lack of Support* | Mothers emotional exhaustion due to lack of structural systems to support them and the pressure of the dominant cultural narrative of a self sacrificing mother. | *“I couldn't concentrate or I couldn't be with them. I felt like playing with them, but I couldn't. Because I was so emotionally broken. I couldn't connect my brain, you know, to what is happening or I was lost. I was just sitting alone or whiling around.”* |
| *Suppression and Silence* | Mothers conditioned emotional expression of maternal rage as silent, implicit and suppressed. | *“And then you stop complaining because nobody's listening to you. So those complaints stay with you,and you become sadder.”* |
| *Intergenerational Scripts* | Patriarchal scripts rooted in the cultural nuances that are followed by generations and perpetuate an imbalance of power between the mother-in-law and daughter-in-law dynamic. | *“What also makes me really upset is, you know, not only my mother in law, no, any mother in law was also a mother, right? She was also once 30 years old and she also once had one baby. And, you know, maybe it may have been so isolating for her also. And after all these years, you just come back and do the same thing to me.”* |

**Supplementary Table 1.** Offers a transparent presentation of the key themes that emerged from the participant’s narratives. Each theme is accompanied by a concise description and a quote that grounds the findings in the lived experiences of the mother’s that were interviewed.
