# Supplementary figures and images for: Mothering on the edge: exploring maternal anger through feminist psychoanalysis and socio-ecological inequities of two mothers in urban India
Source: Front Glob Womens Health. 2026 Apr 17;7:1751532. doi: 10.3389/fgwh.2026.1751532 (PMC13132850; doi:10.3389/fgwh.2026.1751532)

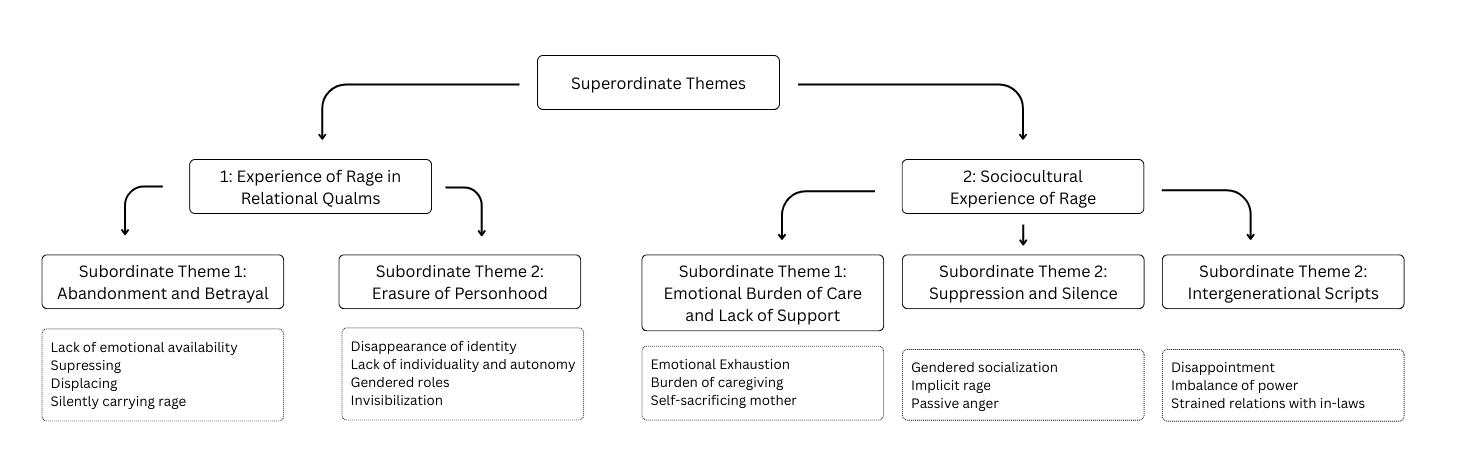

Supplement: Supplementary file 2 [file Image1.png]
